# miRNAs Can Affect Intestinal Epithelial Barrier in Inflammatory Bowel Disease

**DOI:** 10.3389/fimmu.2022.868229

**Published:** 2022-04-13

**Authors:** Xiangjun Xiao, Xiangbing Mao, Daiwen Chen, Bing Yu, Jun He, Hui Yan, Jianping Wang

**Affiliations:** Institute of Animal Nutrition, Sichuan Agricultural University, Key Laboratory for Animal Disease-Resistance Nutrition of China Ministry of Education, Key Laboratory of Animal Disease-Resistant Nutrition and Feed of China Ministry of Agriculture and Rural Affairs, Key Laboratory of Animal Disease-Resistant Nutrition of Sichuan Province, Chengdu, China

**Keywords:** miRNA, inflammatory bowel disease, intestinal epithelial barrier, gene targets, signaling pathways

## Abstract

The most obvious pathological characterization of inflammatory bowel disease (IBD) is intestinal epithelium erosion and severe inflammation invasion. Micro-ribonucleic acids (miRNA or microRNA), single-stranded noncoding RNAs of ~22 nucleotides, have been considered as the potential therapeutic targets in the pathogenesis of IBD. Many previous studies have focused on the mechanisms that miRNAs use to regulate inflammation, immunity, and microorganisms in IBD. The review highlights in detail the findings of miRNAs in the intestinal epithelial barrier of IBD, and focuses on their gene targets, signaling pathways associated with IBD, and some potential therapies. It will be beneficial for the elucidation of the interaction between miRNAs and the intestinal epithelial barrier in IBD and provide a theoretical reference for preventing and treating IBD in the future.

## 1 Introduction

The intestine is an extremely important organ in mammals. Besides its responsibility of providing nutrients to other organs, tissues, and cells throughout the body *via* its digestive and absorptive functions, it is the first barrier of the body that can help maintain intestinal homoeostasis (1). The intestinal epithelial barrier defects can lead to a series of problems, including immune dysfunction, pathogen invasion, and over-pro-inflammatory cytokine secretion. Over the past years, the incidence of inflammatory bowel disease (IBD) has rapidly risen in Europe, the United States, and Asia (2).

IBD, including Crohn’s disease (CD) and ulcerative colitis (UC), is a chronic non-specific disease derived from intestinal epithelium erosion and inflammation invasion that has a serious impact on the life quality of patients (1). Unfortunately, the medical field has not come up with effective and feasible treatment methods because of the recurrent and persistent features of IBD. Researchers have established various IBD models, such as cell models [tumor necrosis factor (TNF)-α and lipopolysaccharide (LPS) induction] (3) and mouse and rat models [adherent-invasive *Escherichia coli* (AIEC) challenge, interleukin (IL)-10 knockout, and dextran sulfate sodium salt (DSS) and trinitro-benzene-sulfonic acid (TNBS) administration] (4–6). Recent studies have shown that there is a relationship between IBD and micro-ribonucleic acid (miRNA or microRNA) (3, 5, 7–10).

In 1993, Lee et al. discovered the miRNA for the first time, which is Lin-4 in *C. elegans* (11). Among the latest data provided by miRBase, 48,860 different mature miRNAs have been found in 271 representative organisms, and the human genome encodes 2,654 mature miRNAs (12). miRNAs are a class of ~22 nucleotides evolutionally conserved through small noncoding RNAs. Their function is to negatively regulate target messenger RNA (mRNA) expression by binding to their 3’-untranslated regions (3’UTR), leading to mRNA degradation, or directly induce translational suppression. To a certain extent, miRNAs could regulate cell proliferation, differentiation, apoptosis, and metabolism (13). According to recent studies, their results suggested that miRNAs could have a negative or positive affect on the occurrence and development of IBD (1, 4, 5, 7, 14–17). Here, we review relevant miRNAs in IBD diagnosis, relationship of miRNA and intestinal epithelial barrier in IBD (including the targets, function, and pathway of miRNA), and prospective miRNA therapies to IBD.

## 2 The Relevant miRNAs in IBD Diagnosis

There is no gold standard for the diagnosis and monitoring of IBD. The usual diagnosis is based on clinical manifestations and endoscopy with histopathological examination (18). Archanioti et al. (2011) have discussed the differential expression of some miRNAs as non-invasive and inexpensive biomarkers in UC and CD (19). It is conducive to discrimination between UC and CD, prognostic evaluation and early decisions of IBD, and monitoring and guidance of ongoing treatment and postoperative prognosis and recurrence (20). In this section, we summarized researches over the past decade and found miRNA alteration between UC and CD patients from their intestine biopsies, feces, and peripheral blood (**Table 1**).

**Table 1:** miRNA biomarkers in IBD.

Sample types	Expression	miRNAs	References
**UC vs. normal**			
Intestine biopsies	Increase	miR-15*, miR-16, miR-19a, miR-21, miR-31, miR-31-3p*, miR-101, miR-125b*, miR-206*, miR-223, miR-155, miR-594	(1, 7, 17, 21–24)
	Decrease	miR-141, miR-200b, miR-214-3p*, miR-429	(25, 26)
Feces	Increase	miR-16-5p, miR-21-5p*, miR-223*, miR-1246*	(27, 28)
	Decrease	N/A	
Peripheral blood	Increase	miR-16, miR-21, miR-28-5p*, miR-106a, miR-151-5p*, miR-155, miR-199a-5p, miR-362-3p	(29, 30)
	Decrease	N/A	
**CD vs. Normal**			
Intestine biopsies	Increase	miR-16, miR-19a, miR-21, miR-30c*, miR-101, miR-223, miR-130a*, miR-146a, miR-155, miR-594	(1, 6, 7, 31)
	Decrease	miR-141, miR-200a*, miR-200b, miR-200c,* miR-429	(25)
Feces	Increase	miR-15a-5p*, miR-16-5p, miR-24-3p*, miR-27a-3p*, miR-128-3p*, miR-142-5p*, miR-223-3p, miR-223-5p, miR-3074-5p*	(27, 32)
	Decrease	miR-10a-5p*, miR-10b-5p*, miR-141-3p*, miR-192-5p*, miR-200a-3p*, miR-375*, miR-378a-3p, and let-7g-5p*	(32)
Peripheral blood	Increase	miR-16, miR-21, miR-23a*, miR-29a*, miR-106a, miR-107*, miR-126*, miR-155, miR-191,* miR-199a-5p, miR-200c*, miR-362-3p, miR-532-3p*	(29, 33)
	Decrease	N/A	

^*^Possible differential miRNAs between UC and CD.

### 2.1 Intestine Biopsies

At present, intestinal biopsies are the most important sample types for researchers to analyze the differential expression of miRNAs between UC and CD patients. Fang et al. (2018) found that miR-31-3p was significantly elevated in UC biopsies rather than CD biopsies compared with controls (21). The alteration of miRNA expression was not confined to the inflamed position, but already pre-existed in the non-inflamed position (19). Nguyen et al. (2014) have also verified such a standpoint; miR-30c and miR-130a expression was upregulated in both inflamed and non-inflamed ileal CD biopsy specimens compared to controls (6). Moreover, there was no significant difference in miR-30c and miR-130a expression between the inflamed and non-inflamed ileal CD group, and between UC patients and healthy controls (6). By comparing with healthy controls, miR-31 expression of sigmoid colon biopsies is strongly upregulated in active UC (~11-fold) and more moderately upregulated in inactive UC (17). Thus, it may be possible to distinguish active or inactive IBD though analyzing the level of miRNA expression. miR-146a and miR-155 were upregulated in the inflamed duodenal mucosa from CD patients compared to controls (31). However, a report indicated that the expression of 7 miRNAs (miR-16, miR-19a, miR-21, miR-101, miR-155, miR-223, and miR-594) was elevated in the colonic mucosa of both UC and CD compared to healthy controls (1). In the inflamed intestine biopsies of both active UC and CD, Tian et al. (2019) observed that miR-31 was upregulated (7). miR-15 (22), miR-125b (23), and miR-206 (24) were upregulated in colonic tissue of active UC patients compared with healthy controls, but the authors did not compare the difference of the above 3 miRNAs between CD and controls. In addition, compared to healthy controls, Zidar et al. (2016) identified that 3 miRNAs (miR-141, miR-200b, and miR-429) were decreased in the colonic mucosa of both CD and UC, and only 2 miRNAs (miR-200a and miR-200c) were decreased in the colonic mucosa of CD (25). miR-214-3p was downregulated in sigmoid colonic biopsies of active UC patients compared with healthy controls (26).

### 2.2 Feces and Peripheral Blood

For intestinal mucosal samples, the acquisition of feces and peripheral blood from UC or CD patients is a relatively non-invasive method.

(1) Feces: Evaluating fecal miRNA expression profiles of IBD patients and healthy controls *via* microarray analysis, Zhou et al. (2021) found that the increase of miR-21-5p was only observed in the feces of UC patients, and there was a significant increase in miR-16-5p expression in the feces of both UC and CD patients compared with healthy controls (27). There was a significant increase in expression level of miR-223 and miR-1246 in the feces of active UC compared with controls, and only miR-223 was upregulated in the feces of active UC compared with inactive UC. Additionally, miR-223 and miR-1246 had no differences in the feces and peripheral blood between CD and controls, and between active CD or inactive CD (28). Wohnhaas et al. (2020) found that 9 miRNAs (miR-15a-5p, miR-16-5p, miR-128-3p, miR-142-5p, miR-24-3p, miR-27a-3p, miR-223-3p, miR-223-5p, and miR-3074-5p) were significantly upregulated but 8 miRNAs (miR-10a-5p, miR-10b-5p, miR-141-3p, miR-192-5p, miR-200a-3p, miR-375, miR-378a-3p, and let-7g-5p) were significantly downregulated in the feces of CD (32).

(2) Peripheral blood: Paraskevi et al. (2012) found significantly higher levels of miR-16, miR-23a, miR-29a, miR-106a, miR-107, miR-126, miR-191, miR-199a-5p, miR-200c, miR-362-3p, and miR-532-3p in the blood from active CD patients compared to healthy controls (29). The levels of miR-16, miR-21, miR-28-5p, miR-151-5p, miR-155, and miR-199a-5p were increased in the blood from active UC patients compared to healthy controls (29). Omidbakhsh et al. (2018) reported that the expression level of miR-106a and miR-362-3p was increased in the peripheral blood from CD and UC patients compared to healthy controls, and there were significant differences of miR-362-3p in active UC relative to inactive UC (30).

After comparing miRNAs in different tissues between UC or CD patients and control, we found that there are both common miRNAs (i.e., miR-16, 21, 31, 155, and 223) and some differential expressed miRNAs in intestinal biopsies, feces, and peripheral blood. These differential expressed miRNAs could help to distinguish UC and CD in clinical diagnosis. Moreover, a miRNA expression would not be expected to be the same as that seen in intestinal biopsies due to miRNAs of peripheral blood possibly reflecting expression in circulating white blood cells (19). Differences in miRNA composition and expression levels may also be caused by the different evolutionary stage of IBD.

## 3 miRNAs and Intestinal Epithelial Barrier in IBD

In addition to studying abnormal miRNAs in IBD, researchers also analyzed the relationship between miRNAs and intestinal epithelial barrier, in order to find their targets, functions, and related mechanisms (**Table 2**).

**Table 2:** Summary of specific miRNAs and its target gene involved in IBD model.

miRNAs	IBD model	Expression	Direction	Target genes	References
miR-21	DSS-induced mice	Knockout	−	*RhoB*	(5)
miR-29a	DSS-induced mice	Increase	+	*ATG9A*	(10)
miR-30c miR-130a	AIEC-infected mice	Increase	+		(6)
miR-31	DSS or TNBS-induced mice	Decrease	+	*IL17RA*	(7)
	Colon tissue isolated from UC	Increase	+	*TLSP*	(17)
miR-106a miR-106b	HCT116 cells	Increase	+	*ATG16L1*	(34)
miR-122a	TNF-α-treated Caco-2 cells	Increase	+	*Occludin*	(14)
miR-122b	TNF-α-treated Caco-2 cells	Increase	−	*MLCK, c-Jun*	(35)
miR-126	DSS-induced mice	Decrease	−		(9)
miR-155	TNF-α and LPS-treated IMF isolated from UC	Increase	+	*SOCS1*	(3)
miR-200b	TGF-β1-induced IEC-6	Increase	−	*ZEB1, SMAD2*	(36)
miR-206	HT-29 cells and DSS-induced mice	Increase	+	*A3AR*	(24)
miR-214-3p	HT-29 cells	Decrease	+	*STAT6*	(26)
miR-223	TNBS-induced mice	Increase	+	*Claudin-8*	(1)
miR-375	Colon tissue isolated from UC and CD	Decrease	+	*TLR4*	(15)

“+” represents promotion; “−” represents suppression; UC, ulcerative colitis; CD, Crohn’s disease; DSS, dextran sodium sulfate; TNBS, 2,4,6-trinitrobenzene sulfonic acid; IEC, intestinal epithelial cells; TGF-β1, transforming growth factor β1; AIEC, adherent-invasive Escherichia coli.; TNF-α, tumor necrosis factor α; LPS, lipopolysaccharide; IMF, intestinal myofibroblasts; A3AR, A3 adenosine receptor; STAT, signal transducer and activator of transcription.

### 3.1 miRNAs and the Integrity of Intestinal Epithelial Cells

The integrity of IECs underpins the intestinal epithelial barrier. miRNAs are also a special factor to influence intestine mucosal structure in IBD. For example, the negative effects of miR-126 knockdown were observed in DSS-challenged mice, which manifested as incomplete mucosal epithelium and ulcer of colon tissue (9). In addition, miRNAs have the ability to promote the epithelial regeneration after injury. An *in vitro* study demonstrated that with endogenous over-expression of miR-200b, IEC-6 cells have a potential for proliferation through promoting the protein expression of cyclin D1 (36). miR-31 regulates mucosal repair response *via* the Wnt signaling pathway and modulates the expression of target genes related to epithelial regeneration [thymic stromal lymphopoietin (*TSLP*) and ras homolog family member A (*RhoA*)] (7, 17, 21).

Importantly, miRNAs mediate the programmed cell death of IECs, which will regulate gut integrity in IBD. Shi et al. (2013) demonstrated that miR-21 knockout decreased a higher number of apoptotic epithelial cells in colitis mice (5). Meanwhile, miR-21 targets a variety of apoptotic genes, including programmed cell death 4 (*PDCD4*) and cell division cycle (*Cdc24*), to control the cell cycle progression (5, 37). Autophagy, named type II programmed cell death, is a protective mechanism to tightly regulate homeostasis (38). The *in vivo* study indicated that AIEC infection reduced the levels of autophagy-related gene 5 (*ATG5*) and *ATG16L1*, and then inhibited autophagy *via* upregulating miR-30c and miR-130a, which led to inflammatory reaction deterioration in mouse IECs (6). Consistent with the above results, ileal samples from CD patients also have increased levels of these same miRNAs and reduced levels of autophagy-related proteins (6). In HT29 and HCT116 cells and DSS-induced colitis of mice, Xu et al. (2018) found that over-expression of miR-29a suppressed the production of autophagy spots and the expression of *ATG9A* (10). Moreover, miR-106a and miR-106b can also inhibit the expression of genes responsible for autophagy pathway in IECs, consequently participating in the development of IBD (34).

The junctional complexes, including tight junctions (TJs), adherens junctions (AJs), and desmosomes, create a paracellular pathway that only allows the passage of certain solutes and fluids (39). Knockdown of TJ proteins [such as claudin and zonula occludens (ZO)-1] significantly reduces transepithelial resistance (TEER), proving that TJ proteins are necessary to maintain normal intestinal epithelial barrier properties (1). Defects in intestinal TJ barriers elicit an increase in intestinal permeability and antigenic penetration, contributing to the development of IBD (1, 14).

The miRNA expression levels of intestinal mucosa in IBD patients can significantly affect intestinal TJ barriers. As observed in the colon of human UC patients and IL-10 knockout mice, the high levels of miR-21 had also been detected in the colon tissues of DSS-induced colitis mice (5, 16). After DSS treatment, miR-21 knockout mice presented lighter levels of intestinal histopathological alteration (such as paracellular permeability and epithelial erosions) when compared with WT mice (5). Moreover, Yang et al. (2013) found that upregulated miR-21 led to the degradation of *RhoB* mRNA and then the downregulating occludin expression and increasing permeability in Caco-2 cells (16). In TNF-α-treated Caco-2 cells, over-expression of miR-122a can deplete occludin in the manner of binding to occludin 3’UTR and further destroy the paracellular permeability (14). *Via* immunofluorescence analysis to TJ proteins, ectopic expression of miR-200b preserves claudin-1 and ZO-1 levels to rescue the integrity of TJ morphology and attenuates the intestinal TJ barrier dysfunction in TNF-α-treated Caco-2 cells (35). Additionally, in the gut mucosa of IBD patients, miR-375 was downregulated with the increasing intestinal permeability (15).

### 3.2 miRNAs and Intestinal Myofibroblasts

Besides IECs and junctional complexes, there are mounts of fibroblasts in the lamina propria below the gut mucosal epithelium. Intestinal fibrosis is the phenotype of IBD complication. Its pathological manifestation is an excess of extracellular matrix (ECM) secreted by intestinal myofibroblasts (IMFs) and eventually deposited in the intestine (40). This may cause serious consequences, i.e., narrow intestine lumen, poor nutrients, and even forming ileus. In patients with CD, the known abnormal deposition of ECM occurs in all anatomical layers of the entire intestine, which is different from that of patients with UC (25).

Exogenous supplementation of miR-29b reduced the level of collagen induced by transforming growth factor (TGF)-β1, and may be a candidate approach to relieve intestinal fibrosis of patients with CD (41). As a key cell population contributing to mucosal damage in IBD, IMF of patients with UC induced by inflammatory mediators (TNF-α and LPS) can stimulate miR-155 levels, and miR-155 also induces the production of cytokines (IL-6 and IL-8), which leads to the transmitting inflammation, and then aggravates the intestinal epithelium inflammation (3).

Epithelial-to-mesenchymal transition (EMT) is deemed to be a contributor to the pool of activated fibroblasts in fibrosis of IBD (25). The miR-200 family are typical miRNAs that suppress EMT formation. However, miR-200b showed stronger anti-EMT function than miR-200a and miR-200c in various studies (25, 36, 42, 43). In TGF-β1-mediated EMT of IEC-6, miR-200b supplementation had a significant alleviation of EMT and fibrosis through depleting target genes [zinc finger E-box-binding protein (*ZEB*)*1*, *ZEB2*, and *SMAD2*], decreasing vimentin and α-smooth muscle actin, and increasing E-cadherin (36, 43). The alteration of these genes will induce intestinal fibrosis, and eventually disrupt the junctional complexes of epithelial cells and the epithelial barrier functions.

### 3.3 miRNAs and Immune Function of IECs

IECs can regulate immune response and then maintain intestinal barrier health. On one hand, IECs respond to cytokines secreted by immune cells through numerous cell surface receptors. On the other hand, IECs also produce cytokines to modulate intestinal mucosal immunity.

miR-31, as a middle emissary, may react with extracellular pro-inflammatory signals, and directly suppress *IL17RA* in the colonic epithelium of DSS-challenged mice (7). IL-23, as a pro-inflammatory cytokine secreted by IECs, can trigger a series of inflammatory cascades and then lead to intestinal inflammation, such as IBD. Wang et al. (2016) identified the cross-talk mechanism between the IL-23 pathway and the intestinal epithelial barrier in IBD and showed that miR-223 was a crucial pro-inflammatory component of the IL-23 pathway in IBD patients and animals, and negatively modulated its targets, including claudin-8 (1). Interestingly, Wang et al. (2016) also screened miRNAs from the IBD patients and found that some miRNAs, including miR-223, miR-21, miR-155, miR-19a, miR-101, miR-594, and miR-16, were upregulated (1). However, it is unknown whether other IL-23/miRNAs/Claudin pathways contributed to the onset and development of IBD, which remains to be further studied.

### 3.4 miRNAs and Intestinal Microbiota

The intestinal microbiota encompasses viruses, bacteria, and fungi, which are tightly regulated by the intestinal epithelial barrier in the completion of a series of physiological and biochemical functions of the host. Once this interaction is dysregulated, it may lead to IBD. By comparing fecal miRNA profile between germ-free and conventional mice, Viennois et al. (2019) revealed the existence of interactions between the 12 miRNAs and specific microbiota members (44). In addition, some researchers supported the view that miRNA can be modulated by microbiota to impact the intestinal barrier *via* miRNA expression modulation (45, 46). A high prevalence of AIEC abnormally colonizing the ileal mucosa and invading IECs in CD patients had occurred. Larabid et al. (2020) analyzed the dysregulated miRNAs and their potential target genes in AIEC infection of Caco-2-cl1 cells, and downregulated miR-31c-1-3p may target mucins (*MUC1)* and claudin (*CLDN*) to disturb the intestinal barrier function of IBD patients (47). Moreover, a previous study had indicated that AIEC reduced expression of proteins required for autophagy through upregulating miR-30c and miR-130a in T84 cells and mouse enterocytes, thus conducting self-replication (6). However, probiotic (*Saccharomyces boulardii*) treatment increased bacterial diversity and improved the epithelial integrity proteins (i.e., ZO-1, MUC1, MUC2, and OLDN) in DSS-induced mice, possibly due to inhibiting the expression of pro-inflammatory miR-155 and 233 (48). Another study supported the view that *Clostridium butyricum* improved intestinal epithelial integrity *via* increasing miR-200c expression (4).

## 4 miRNAs and Pathogenesis-Related Signaling Pathways in IBD

When you put all above studies together, the damaged IECs, broken junctional complexes, and abnormal intestinal epithelial fibrosis and immune response are important reasons contributing to the development of IBD. Besides disrupting junctional complexes after directly binding 3’UTR TJ proteins (1, 14), miRNAs can influence some signaling pathways to positively or negatively modulate the function of intestinal epithelial barrier in IBD.

### 4.1 The MLCK/p-MLC Signaling Pathway

**Figure 1:**
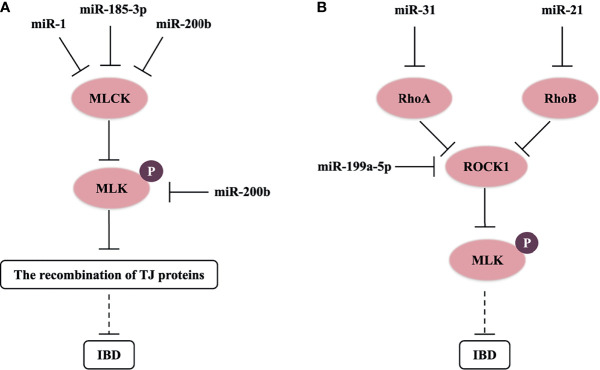
miRNAs control MLCK/p-MLC and Rho/ROCK/p-MLC signaling pathway in IBD. **(A)** miR-1, miR-185-3p, and miR-200b target and inhibit MLCK or p-MLC levels to block the recombination of TJ proteins, and possibly reverse the impaired intestinal barrier function in IBD. **(B)** miR-21, miR-31, and miR-199a-5p respectively target and downregulate RhoB, RhoA, and ROCK1 levels, and possibly inhibit the progress of IBD.

The actin cytoskeleton can control the assembly and function of epithelial TJs under the influence of various stimuli, such as inflammatory cytokines (TNF-α), microorganisms, and RNA (5, 16, 35). Myosin light chain kinase (MLCK) induces contraction of perijunctional apical actomyosin ring in response to phosphorylation of myosin light chain (MLC); thereby, it affects the recombination of TJ proteins and perijunctional actin, and increases the permeability of paracellular pathway (39). Previous studies have revealed that the increasing level of MLCK and MLC phosphorylation (p-MLC) was an important pathway leading to intestinal TJ barrier malfunction in the *in vivo* and *in vitro* models of IBD (**Figure 1**) (35, 49). Moreover, MLCK expression was also upregulated in intestinal biopsy tissue of CD and UC patients (13). Further studies showed that miR-200b targets and inhibits MLCK and p-MLC levels to block TJ protein redistribution in TNF-α-treated Caco-2 cells (35). In addition, miR-185-3p and miR-1 can downregulate MLCK expression to reverse the impaired intestinal barrier function in the IBD model (50, 51).

### 4.2 The Rho/ROCK Signaling Pathway

Rho-associated kinase (ROCK) of guanosine triphosphate hydrolase enzymes (GTPases), known as a key downstream molecule of the Rho family in intracellular actin signaling, is also a crucial kinase to activate phosphorylation of MLC. In the pathological process of IBD, the Rho/ROCK/p-MLC pathway will result in alteration of TJ protein structures *via* mediating F-actin cytoskeleton (52). Chen et al. (2016) found that miR-31 downregulated RhoA expression and ROCK/p-MLC pathway, and suppressed the invasion and migration of gastric cancer cells (53). miR-199a-5p can also downregulate ROCK1 expression, and then suppress IBD-related colon cancer (54). Furthermore, RhoB (a molecular switch that turns on or off ROCK) is the target of miR-21, which may result in the depleting level of occludin in UC dysfunctional process and Caco-2 cells (16). Then, we think that miRNAs also have a great opportunity to regulate the structure of TJs through the Rho/ROCK/p-MLC pathway in IBD (**Figure 1**). A recent study showed that the Rho/ROCK/p-MLC pathway also regulated cell contractility in different manners (55). miRNAs could affect cell contractility in IBD, which needs to be further researched.

### 4.3 The NF-κB Signaling Pathway

**Figure 2:**
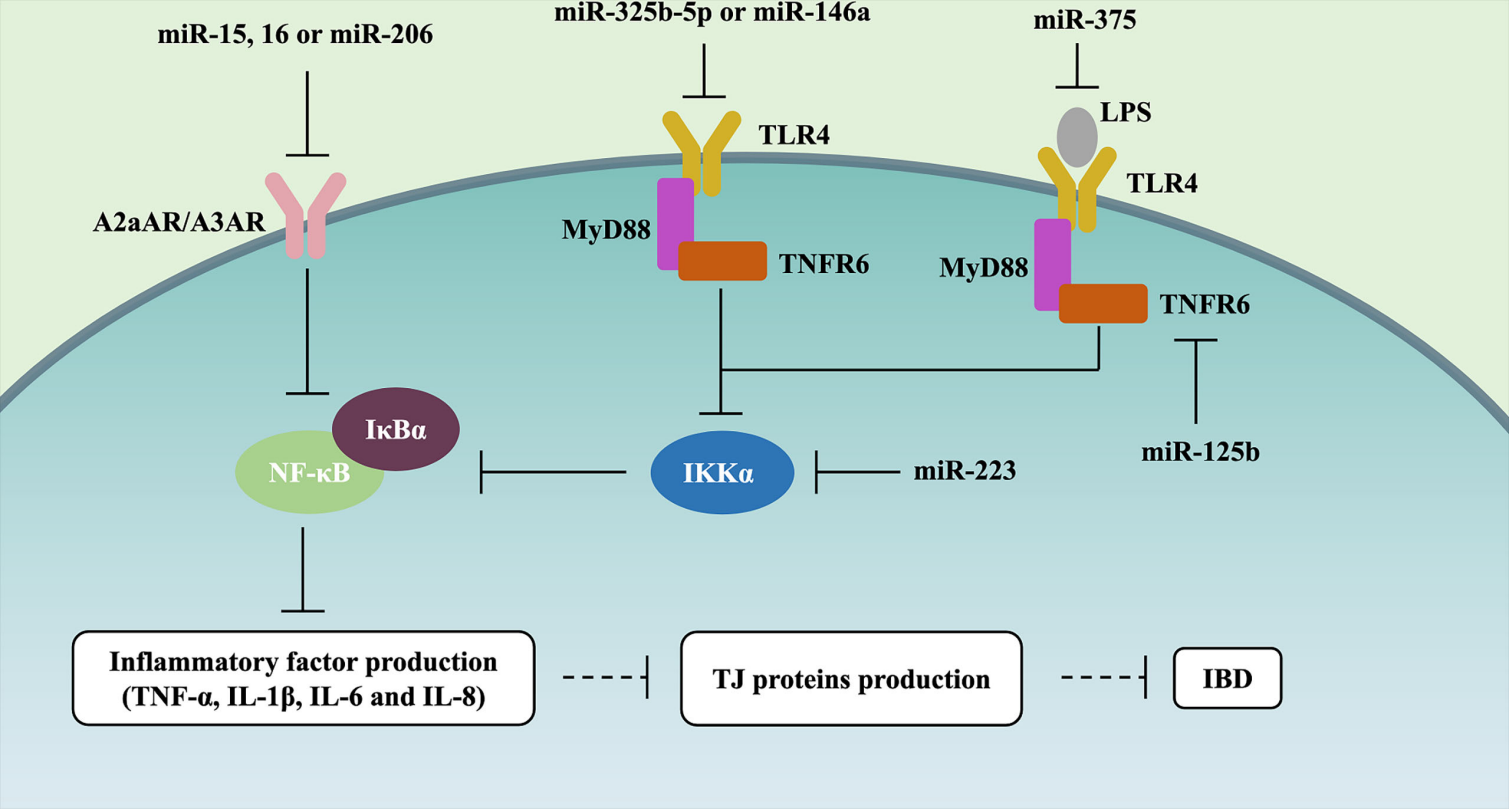
miRNAs control NF-κB signaling pathway in IBD. miR-15, miR-16, and miR-206 downregulate A2aAR or A3AR expression, further inhibiting NF-κB and the level of IL-8 and IFN-γ in UC. miR-323b-5p and miR-146a target and inhibit the TLR4/MyD88/NF-κB signaling pathway. miR-375 competitively inhibits TLR4 expression, further alleviating the generation of pro-inflammatory cytokines (TNF-α, IL-1β, IL-6, and IL-8) and the deterioration of intestinal mucus (i.e., ZO-1 and occluding downregulation). miR-125b and miR-223 inhibit TLR4/MyD88/NF-κB signaling pathway through respectively downregulating TRAF6 and IKKα in IBD.

The Toll-like receptor (TLR)-4/myeloid differentiation factor 88 (MyD88)/nuclear factor-κB (NF-κB) signaling pathway always has abnormal activation, which will result in inflammatory storms, and destroy the structure and morphology of the intestinal epithelium in IBD patients (56, 57). Recently, many studies have described the role of miRNAs on the TLR4/MyD88/NF-κB signaling pathway (**Figure 2**). Qiao et al. (2018) and Wang et al. (2019) reported that miR-323b-5p and miR-146a would target the TLR4/MyD88/NF-κB signaling pathway in UC (22, 56). miR-375 can competitively inhibit TLR4 expression, and knockdown of miR-375 in the LPS-induced caco-2 cells would stimulate the generation of pro-inflammatory cytokines (TNF-α, IL-1β, IL-6, and IL-8) and deteriorate intestinal mucus, such as the decreasing levels of TJ proteins (i.e., ZO-1 and occludin) (15). Interestingly, miRNAs can also influence the synthesis of other regulatory proteins to control the NF-κB signaling pathway. Co-transfection with miR-125b and miR-223 in LPS-induced HT29 cells may cause the activation of NF-κB and higher levels of pro-inflammatory cytokines (i.e., IL-8 and IL-1β), which is due to miR-125b and miR-223 downregulating *TRAF6* and *IKKα*, respectively (23).

The top and bottom of IECs were full of adenosine, which is involved in the endogenous protective response *via* suppressing the NF-κB signaling pathway during IBD mucosal inflammation (58). Various studies have assessed the effect of miRNAs on the adenosine receptor (AR)/NF-κB signaling pathway (**Figure 2**). miR-15, miR-16, and miR-206 downregulated A2a adenosine receptor (*A2aAR*) or *A3AR* expression, further inhibiting NF-κB and the level of IL-8 and IFN-γ in the pathogenesis of UC (22, 24, 59).

Taken together, these studies support the notion that NF-κB should be one of the signaling pathways by which miRNAs affect gut barrier function, and it is involved in 2 pathways, i.e., TLR4/MyD88 and AR (**Figure 2**).

### 4.4 The MAPK Signaling Pathway

Mitogen-activated protein kinase (MAPK) signaling pathway plays a pivotal role in the process of IBD. It includes four branch routes: extracellular regulated kinase (ERK) pathway, c-Jun amino-terminal kinase (JNK) pathway, p38/MAPK pathway, and ERK5 pathway. The function of JNK and the p38/MAPK pathway is the regulation of gene expression in response to extracellular stimuli, which involves inflammation, cell cycle, cell proliferation, and apoptosis. Over-expression of miR-200b protects the function of the intestinal epithelial barrier by suppressing the TNF-α-upregulated JNK/c-Jun/AP-1 signal and IL-8 synthesis levels in Caco-2 cells (35). Bai et al. (2020) show that miR-195 mimics can alleviate the pathological damage and inflammatory responses in colon of TNBS/ethanol-treated rats, which are related to the inhibition of the p38/MAPK signaling pathway (60). In addition, Valmiki et al. (2017) predicted potential targets of dysregulated miRNAs by using *in silico* prediction tools in colonic mucosal pinch biopsies from the inflamed and non-inflamed regions of the same UC patient, and showed that some miRNAs, including miR-125b, miR-155, miR-223, and miR-138, exert an essential effect on the MAPK pathway (61). However, there is still a lack of more reliable data about miRNAs influencing intestinal epithelial function (especially proliferation and apoptosis of IECs) in IBD through mediating the MAPK signaling pathway.

### 4.5 The JAK/STAT Signaling Pathway

STAT (signal transducer and activator of transcription), an important molecule of the Janus kinase (JAK)/STAT signaling pathway, is activated by IL-6 (62). By analyzing colon samples from 24 patients with active UC and 20 healthy persons, Li et al. (2017) showed that miR-214-3p had a lower level in patients with UC, which could be involved in the pathogenesis of UC through targeting STAT6 (26). In a mouse model of UC, miR-495 treatment downregulated STAT3 expression and further inhibited the JAK/STAT3 signaling pathway, which promoted IEC proliferation and claudin-1 level (63). Moreover, the positive feedback loop of STAT3/miR-29a-5p amplifies the effects of chronic inflammation in the IL-6-stimulated IECs, and leads to colon cancer development (62).

Besides directly regulating STAT expression, miRNAs can also influence gut barrier function in IBD *via* affecting suppressors of cytokine signaling (SOCS) level. It is well known that SOCS is deemed as a STAT negative regulator and may suppress the development of malignant disease (64). Epigenetic silencing of SOCS3 expression contributes to fibrosis in CD because of a miR-19b-mediated inhibition mechanism (65). Pathak et al. (2015) reported that miR-155 inhibited SOCS1 expression and increased pro-inflammatory cytokine production in LPS-induced IMF (3). However, as regards the connection among miRNA, SOCS, and STAT, it is still unclear in fibrosis of IBD.

### 4.6 The TGF-β Signaling Pathway

**Figure 3:**
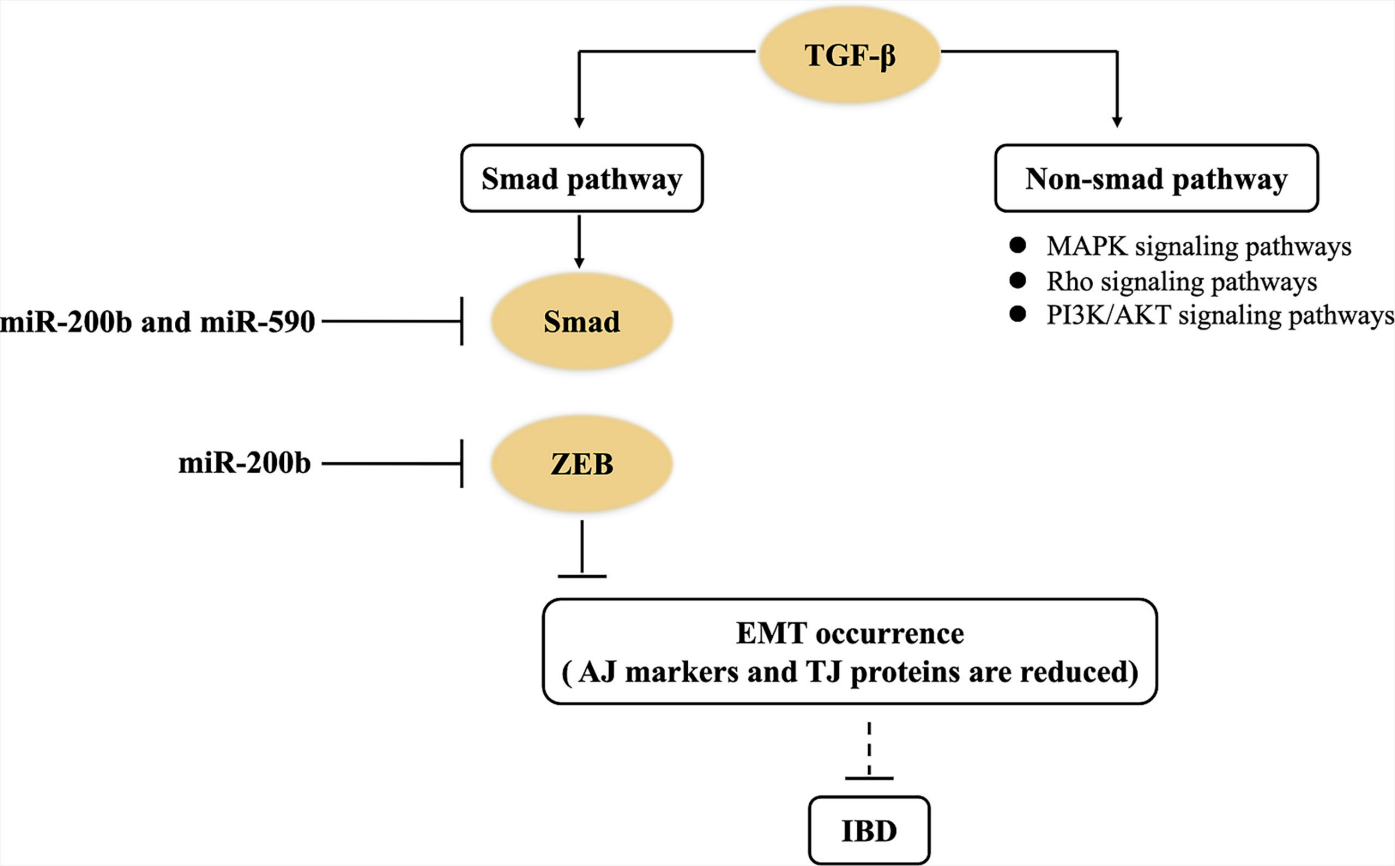
miRNAs control the TGF-β signaling pathway in IBD. miR-200b downregulates the expression of target genes (including Smad2, ZEB1, and ZEB2) to inhibit EMT occurrence in the TGF-β1/Smad signaling pathway of IBD. miR-590 negatively regulates Smad2 and Smad3 in the non-Smad-dependent TGF-β signaling pathway of IBD.

TGF-β is a pleiotropic cytokine. The activation of the TGF-β signaling pathway contributes to alleviate inflammation reaction, but its excessive activation will accelerate tissue fibrosis (31, 66). TGF-β signaling pathways are generally divided into Smad dependence and non-dependence. The former shows that TGF-β binds to type I and type II receptors (RI and RII) on cell membranes and form functional complexes, and then RI recruit downstream Smad protein, which induces the aggregation of Smad protein in the nucleus (67). At the same time, Smad in the nucleus trans-activates two major transcription factors (i.e., ZEB and Snail), which eventually achieve EMT occurrence. In the course of EMT formation, epithelial AJ markers (e.g., E-cadherin) and TJ proteins are reduced, and interstitial cell markers (e.g., α-SMA and vimentin) are increased, which subsequently transform into EMT to promote intestinal fibrosis (36). miRNAs, serving as one of the most important regulators, can be involved in the intestinal fibrosis of IBD through the TGF-β signaling pathway (**Figure 3**). miR-200 and miR-29 families are typical anti-fibrosis miRNAs in intestine (36, 41, 43). In an *in vitro* experiment, miR-200b directly inhibited the expression of target genes (including *Smad2*, *ZEB1*, and *ZEB2*) in the TGF-β1/Smad signaling pathway (36, 43). Naghdalipour et al. (2020) have shown that miR-590 was negatively correlated with Smad2 and Smad3 in the TGF-β signaling pathway of UC patients (66). However, there are little data to prove that miRNA can affect the state of intestinal fibrosis by regulating Snail expression in IBD.

In addition, non-Smad-dependent pathways activated by the TGF-β receptors include various branches of MAPK signaling pathways, Rho signaling pathways, and PI3K/AKT signaling pathways (67). These non-Smad pathways can induce dissolution of AJ proteins (e.g., E-cadherin) and TJ proteins (e.g., occludin), and control EMT (68). Nowadays, there are few studies about how miRNAs regulate TGF-β-induced non-Smad-dependent pathways in IBD. Therefore, there are still many questions on how miRNAs regulate intestinal fibrosis of UC patients through the TGF-β signaling pathway.

### 4.7 miRNAs, Apoptosis, and Autophagy in IBD

Apoptosis has three main pathways, namely, extrinsic pathways (death receptor pathway of apoptosis), intrinsic pathways (mitochondrial pathway of apoptosis), and the endoplasmic reticulum (ER) pathway. The Bcl-2 family are typical apoptosis genes including pro-apoptotic genes (e.g., *Bax*, *Bak*, and *Bcl-Xs*) and anti-apoptotic genes (e.g., *Bcl-2*, *Bcl-XL*, *Bcl-W*, and *Mcl-1*).

Recent studies suggest that miRNA-mediated apoptosis can improve or deteriorate the development of intestinal epithelial function in IBD. Bian et al. (2011) and Chen et al. (2020) revealed similar results that miR-150 or miR-16 can downregulate the expression of *Bcl-2* to disrupt colonic epithelium in DSS-induced mouse IBD models (69, 70). TGF-β may downregulate miR-29b expression, which will inhibit the gene expression of *IL-6/Mcl-1* (an anti-fibrotic mediator) or the *IL-8*/*Mcl-1* axis, and then lead to increased collagen deposition and promote intestinal fibrosis in patients with CD (71). However, there are still two questions: (1) the authors determined *Mcl-1* as the target of miR-29b, but did not explain why *IL6* or *IL-8* surmounts miR-29b to direct downregulation of *Mcl-1*; (2) the author brought up the idea that Mcl-1 is an anti-apoptotic gene, but did not clearly describe its corresponding role in CD intestinal fibrosis. Li et al. (2017) analyzed miR-665 expression in 89 freshly isolated IBD samples and DSS-treated colonic mucosal tissues, and found that the upregulated miR-665 promoted apoptosis of IECs through inhibiting ER stress regulators (*XBP1* and *ORMDL3*) (72). In summary, it is found from the above results that the intestinal epithelial damage of IBD is mostly formed by miRNAs regulating anti-apoptotic proteins and ER stress. However, the related mechanisms need to be further studied.

The miRNA-mediated autophagy plays an important role in intestinal epithelial barrier function in IBD. Autophagy, known as a self-protective process, is the eukaryotic cellular response to stimuli, such as starvation, hypoxia, other external pressures, and inflammatory factor. The process delivers cytoplasmic cargo (e.g., organelles and cytoplasmic macromolecules) to the lysosome through a double membrane-bound vesicle, and then fuses with the lysosome to form an autolysosome for degradation and recycling (73). Wang et al. (2018) have summarized that miRNAs mainly regulate autophagy *via* three ways in IBD: (1) miRNAs target autophagy-related genes, such as *ATG5*, *ATG16L1*, *ATG9A*, *NOD2*, and *IRGM*, and thereby modulate intestinal epithelial function; (2) miRNAs regulate the unfolded protein response (UPR) during endoplasmic reticulum stress to involve autophagy; (3) miRNAs can regulate autophagy *via* controlling NF-κB and mTOR signaling pathways and affecting anti-inflammatory or proinflammatory effects (2). Lin et al. (2018) also showed that the upregulated miR-143 targeted ATG2B to inhibit autophagy, and reduced IκBα levels (74).

## 5 miRNA-Related Potential Therapies to IBD

Due to the chronic, recurrent, and lifelong characteristics of IBD, there is currently no possibility of a complete cure. In this section, we summarized relevant studies and showed that three potential therapies (namely, herbal and plant, probiotics, and miRNA manipulation for inhibitors or mimics) could be conducive to IBD (**Table 3**). Nevertheless, the off-target effects become a potential risk because a single miRNA have multiple target genes. To advance the above potential therapies into the clinic, we need to develop preclinical animal experiments to verify and assess feasibility of therapies ahead of time.

**Table 3:** Some beneficial therapeutic agents through targeting abnormal miRNA in IBD models (mice and rats).

Treatments	Therapeutic agents		Outcomes	Model	Dose and Regimen	References
Herbal and plant	Resveratrol		miR-31, Let7a, miR-132 downregulation; increased anti-inflammatory T cell responses	TNBS-induced mice	Dose: daily oral gavage of 100 mg/kg	(75)
	Cinnamaldehyde		miR-21, miR-155 downregulation; inhibition of NLRP3 inflammasome activation	5% DSS-induced mice	Dose: daily oral gavage of 10 mg/kg	(76)
	Mango polyphenolics		miR-126 upregulation, inflammation alleviation	3% DSS-induced rat	Dose: 89.74 mg gallic acid equivalents (GAE)/kg	(77)
	Sinomenine		miR-155 downregulation; inflammation alleviation	5% TNBS-induced mice	Dose: daily oral gavage of 100 mg/kg or 200 mg/kg	(78)
Probiotics	*Clostridium butyricum*		miR-200c upregulation, decreased the transepithelial permeability, inflammation alleviation	TNBS and AOM-induced mice	Unclear	(4)
miRNA manipulation for inhibitors or mimics		miR-7a-5p antagomir	p-JNK downregulation, ZO-1 upregulation	TNBS-induced mice	Dose: 100 nmol/kg, 2 h after TNBS treatment; tail vein injection	(79)
		miR-16 antagomir	Bcl-2 upregulation	3% DSS-induced mice	Dose: 5 mg/kg, twice a week; IP administration	(69)
		miR-31 mimics	Increased body weight and colon length, epithelial cell proliferation promotion	3.5% DSS-induced miR-31 KO mice	Dose: OKGM-PS-MIR31 microspheres (150 ml, 21 mg/ml), enema administration	(7)
		miR-31-3p agomir	RhoA downregulation	2% DSS-induced mice	Dose: 80 μg; days 1, 3, and 5 of DSS treatment, intracolonic administration	(21)
		anti-miR-122a	Occludin upregulation	TNF-α-induced mice, intestinal perfusion model	Dose: pre-miR-122a (25 nM) and lipofectamine (50 μl); lumen of small intestine injection	(14)
		miR-155 antagomir	ZO-1, occludin, claudin-1 upregulation	3% DSS-induced mice	Dose: 100 μl, dissolved in saline at 2 mg/ml, daily for the last 3 days before tissue harvest; IP administration	(80)
miRNA manipulation for inhibitors or mimics		miR-195 agomir	The pathological damage alleviation to the colon	TNBS-induced rat	Unclear	(60)
		miR-223 antagomir	*CLDN8* reactivation	TNBS-induced mice	Dose: 7.5 mg/kg; IP administration	(1)
		miR-223 agomir	Bcl-2 and Bcl-xl downregulation	2.5% DSS-induced mice	Dose: 1.5 mg/kg/day, 24 h after DSS administration; IP administration	(81)

DSS, dextran sodium sulfate; TNBS, 2,4,6-trinitrobenzene sulfonic acid; AOM, acute otitis media; KO, knock out; NLRP3, nucleotide-binding domain and leucine-rich repeat containing Protein 3; IP, intraperitoneal; ZO-1, zonula occluden-1; CLDN8, Claudin-8; RhoA, ras homolog family member A; JNK, c-Jun amino-terminal kinase; TNF-α, tumor necrosis factor α; OKGM, oxidized konjac glucomannan; PS, peptosome.

### 5.1 Herbal and Plant

Herbal and plant therapy, frequently considered as complementary or alternative medicine, is highly used for curing IBD in the world. Resveratrol, cinnamaldehyde, mango polyphenolics, and sinomenine reduce the miR-21, miR-31, miR-126, and miR-155 level of colon to ameliorate experimental colitis, respectively (75–78). Cinnamaldehyde (CA), extracted from the essential oil of cinnamon leaves, is a potential medicine to cure UC in mice; CA at a dose of 10 mg/kg was orally administered once daily for 7 days, which might decrease miR-155 to attenuate the symptoms of DSS-induced colitis, including disturbed disease activity index (DAI), shortened colon lengths, and destroyed intestinal epithelial structure (76). Resveratrol (daily oral gavage of 100 mg/kg), a natural product found in a variety of plant products, also has similar effects in TNBS-induced colitis in mice; that is, resveratrol can reduce miR-31 levels and then improve body weight and colitis symptoms (75). Mango polyphenolics targets miR-126/PI3K/AKT and subsequently inhibits the activity of downstream (NF-κB), which leads to the decrease of inflammatory factors, and then improves the integrity of the intestinal epithelium in DSS-induced colitis of rats (77). Kim et al. (2016) calculated the selected dose of a mango beverage, and it was equivalent to 89.74 mg gallic acid equivalents (GAE)/kg/day for rats (77). *Sinomenine* (at a dose of 100 or 200 mg/kg orally administered once daily for 7 days), a pure alkaloid isolated root of *Sinomenium acutum*, could decrease the level of miR-155 and *TNF-α*, and eventually ameliorate the histological score of colon tissue in TNBS-induced colitis of mice (78). Therefore, we can expect the influence of these herbs and plants on abnormal miRNAs in the intestinal epithelial barrier of IBD.

### 5.2 Probiotics

Probiotics are a good choice in remission of IBD. Butyrate-producing bacteria have an important role in intestinal epithelial barrier integrity because butyrate is the main nutrient for the regeneration and rehabilitation of IECs (82). Xiao et al. (2017) found that *Clostridium butyricum* treatment exhibited inconspicuous inflammation, elongated epithelial microvillus, and increased TER in TNBS-treated mice, and the role of *Clostridium butyrate* in alleviating colitis could be derived from increasing miR-200c expression (4). However, the authors offer no explanation for the distinction between *Clostridium butyricum* and butyrate in regulating miR-200c expression of IBD. Sodium butyrate can enhance miR-200c expression and then downregulate EMT-associated genes in HCT116 (83). Therefore, we believe that butyrate rather than *Clostridium butyrate* is a main substance by regulating the expression of miRNAs to cure IBD.

### 5.3 Use miRNA Manipulation for Inhibitors or Mimics to Treat IBD

According to the mode of action of miRNAs, it could be desirable to replenish miRNA expression (gain of function) or to block miRNAs (loss of function), which could achieve the purpose of alleviating IBD. Gain-of-function strategies (such as applying chemically synthesized miRNA mimics or agomirs) and loss-of-function strategies (such as miRNA-sponge technology or applying miRNA antagomir) have been introduced in detail by Lima et al. (2018) (84).

#### 5.3.1 The Preclinical Evidence of Animal Experiments

At present, these two strategies have been applied to preclinical animal models of IBD, but there is lack of clinical evidence (**Table 3**). For instance, in the DSS-induced miR-31 knockout mice model, the enema administration of peptosome-miR31 mimics encapsulated into oxidized konjac glucomannan contributes to a reduction of inflammatory reaction, increasing body weight and colon length and promoting epithelial cell proliferation compared with controls (7). However, whether miR-31 inhibits or accelerates the progression of IBD remains to be further investigated, as it has been found to be upregulated in the clinic (7, 85). Bai et al. (2020) assessed the role of miR-195 agomir in UC model rats and found that it alleviated the pathological damage to the colon and inflammatory responses, possibly *via* the inhibition of the MAPK signaling pathway (60).

In this review, we discuss the importance of TJ proteins in IBD. Several miRNA inhibitors or mimics (such as miR-7a-5p antagomir, antisense miR-122a, miR-155 antagomir, and miR-223 antagomir) have been identified that could improve TJ protein expression in the UC or CD animal models (1, 14, 79, 80). There was an increase in occludin mRNA level in the intestinal perfusion model, suggesting that antisense miR-122a (complexed with lipofectamine) could have the ability to reverse the increased intestinal TJ permeability in TNF-α-induced colitis mice (14). By using a mouse model of TNBS-induced colitis, Bao et al. (2021) demonstrated that the tail vein injection of miR-7a-5p antagomir can suppress the inflammatory process, with *in vivo* results suggesting that miR-7a-5p increased expression of ZO-1 and promoted the recovery of intestinal mucosa (79). A study by Wang et al. (2016) demonstrated that the intraperitoneal (IP) administration of miR-223 antagomir in TNBS-induced mice reactivated *CLDN8*, in parallel with recovery from colitis (1). Additionally, in the DDS-induced mice, an intraperitoneal (IP) administration of miR-155 antagomir improved the ZO-1, occludin, and claudin-1 expression and maintained the barrier function (80). Fang et al. (2018) indicated that miR-31-3p agomir ameliorated the severity of DSS-induced colitis through downregulating RhoA in mice (21). The above results also indicate that appropriate miRNA inhibitors or mimics that mediate the expression of TJ proteins are beneficial to the treatment of IBD.

On the other hand, apoptosis genes also seem to be promising targets of miRNA manipulation in IBD treatment. it is well known that *Bcl-2* and *Bcl-XL* play a role in inhibiting apoptosis. Chen et al. (2020) indicated that the IP administration of miR-16 antagomir upregulated the Bcl-2 expression and ameliorates intestine function in 3% DSS-induced mice (69). In contrast, Zhang et al. (2021) observed that miR-223 agomir resulted in Bcl-2 and Bcl-xl downregulation and colonic inflammation remission in DSS-induced mice (81). Interestingly, miR-223 has been reported to be pro-inflammatory by Wang et al. (2016) and Valmiki et al. (2020) (1, 23). We speculate that the inconsistent results may be due to the fact that they used different methods to establish the IBD model: including mice, chemical agents (DSS or TNBS), miRNA types (agomir or antagomir), doses, and duration.

#### 5.3.2 How miRNA Inhibitors or Mimics Increase Treatment Efficiency in IBD

In addition, researchers are also studying more approaches to improve the efficiency of miRNA *in vivo* delivery. Suri et al. (2021) had summarized five main approaches of miRNA delivery: lipid carriers (e.g., liposomes and lipid nanoparticles) and polymeric carriers (e.g., cationic carriers such as polyethyleneimine) along with viral vectors, conjugates, and exosomes (86). Ye et al. found that antisense miR-122a complexed with lipofectamine into the small intestine of colitis mice could alleviate the inflammatory response (14). Tian et al. (2019) also found that peptosome-miR31 mimics encapsulated into oxidized konjac glucomannan were more stable than liposome- and polysaccharide-based nanoparticles (7). Among these miRNA vehicles, exosomes seem to be the focus of discussion in recent years. Exosomes, an extracellular vesicle, have been shown to carry biomolecules, including miRNA. Today, it is widely regarded as a natural nanoparticle for drug delivery. Moreover, Moon et al. (2022) reported that the preclinical and clinical studies of drug loaded into exosomes have mainly focused on the field of cancer (87). Exosomes loading miRNA inhibitors or mimics may be a promising tool for the treatment of IBD. Still, Cao et al. (2021) also found that *Enterotoxigenic Bacteroides fragilis* could downregulate exosome-packaged miR-149-3p level in the plasma of IBD patients (88). As mentioned in a review, exosome-based drug delivery remains challenging because of a lack of standardized isolation and purification methods, limited drug loading efficiency, and insufficient clinical-grade production (87). The utility of exosome-packaged miRNA inhibitors or mimics as therapeutics may also be limited given the complex microenvironment in the gut of IBD patients. In the near future, we need to develop cell-derived artificial exosomes or more new biomaterials to package miRNA inhibitors or mimics.

Of course, combination therapy shows great promise as well. Kwapisz et al. (2021) suggested that anti-TNF agents or vedolizumab with ustekinumab may be the ideal combination for IBD through summarizing the efficacy and safety of combination biologic therapy in clinical practice including patients with 14 CD and 1 UC (89). Therefore, it is likely that a similar combination therapy is a good choice - miRNA manipulation for inhibitors or mimics accompanies with the use of other potential therapies including anti-TNF agents, probiotics, herbal and plant agents, vedolizumab or ustekinumab.

## 6 Conclusions

miRNAs acting as a new kind of biomarkers and therapeutic targets could be a good option for curing IBD. We have summarized the role of clinically relevant miRNAs on the intestinal epithelial barrier of IBD, their target genes, functions, and pathways. They can negatively or positively regulate the occurrence and development of IBD. Moreover, we have identified three of the most promising treatments, namely, herbal and plant, probiotics, and miRNA manipulation for inhibitor or mimic. However, many studies are still in the laboratory and have not been extended to clinical practice. Additional *in vivo* studies under clinical conditions and using animal models of IBD should be conducted. We believe that the use of miRNA inhibitor or mimic could be the most efficient promising approach. For IBD, to advance the use of miRNA therapeutics in the clinic, we need to address challenges (e.g., accurate and safe miRNA inhibitor or mimic delivery, miRNA-regulated genes, and gene network and combination therapy) through several animal experiments in the near future.

## Author Contributions

Conceptualization: XM, DC, BY, and JH. Writing—Original Draft Preparation: XX and XM. Writing—Review: DC and HY. Reference Collection: XX and JW. All authors contributed to the article and approved the submitted version.

## Funding

This study was financially supported by the China Agriculture Research System (CARS-35) and a grant from the Science and Technology Support Project of Sichuan Province (2021YFYZ0008).

## Conflict of Interest

The authors declare that the research was conducted in the absence of any commercial or financial relationships that could be construed as a potential conflict of interest.

## Publisher’s Note

All claims expressed in this article are solely those of the authors and do not necessarily represent those of their affiliated organizations, or those of the publisher, the editors and the reviewers. Any product that may be evaluated in this article, or claim that may be made by its manufacturer, is not guaranteed or endorsed by the publisher.
